# Recent advances in the role of plant metabolites in shaping the root microbiome

**DOI:** 10.12688/f1000research.21796.1

**Published:** 2020-02-26

**Authors:** Richard P. Jacoby, Li Chen, Melina Schwier, Anna Koprivova, Stanislav Kopriva

**Affiliations:** 1 Institute for Plant Sciences, Centre of Excellence on Plant Sciences (CEPLAS), University of Cologne, Cologne, 50674, Germany

**Keywords:** synthetic community, GWAS, microbiome, Arabidopsis, plant microbe interactions, exometabolomics, plant, plant metabolites

## Abstract

The last decade brought great progress in describing the repertoire of microbes associated with plants and identifying principles of their interactions. Metabolites exuded by plant roots have been considered candidates for the mechanisms by which plants shape their root microbiome. Here, we review the evidence for several plant metabolites affecting plant interaction with microbes belowground. We also discuss the development of new approaches to study the mechanisms of such interaction that will help to elucidate the metabolic networks in the rhizosphere.

## Introduction

Plants in their natural environment are in constant interaction with diverse microorganisms. Whereas some microbes harm plants and trigger their defense reaction, others are beneficial for plant performance. Therefore, interactions between plant roots and rhizosphere microbiome are critical for plant fitness in an ambient environment. The technical innovations in cultivation of soil microbes and in sequencing technologies resulted in major biological breakthroughs in our understanding of plant microbiota ^1^. The taxonomical composition of root bacterial microbiome is largely stable and is controlled by the soil and by plant genotype ^2– 4^. Indeed, plants produce a plethora of bioactive secondary metabolites and it has often been speculated that these molecules play an active role in shaping the rhizosphere microbiome ^5, 6^. However, this assumption was largely theoretical because relatively few studies defined which specific plant metabolites exert beneficial or antagonistic effects on distinct microbial strains. This situation is beginning to change since several new studies over the last five years have clearly illustrated how the plant microbiome can be shaped by the direct effects of specific metabolites ^7– 9^. The composition of root exudates varies not only among different plant species but also within different natural populations of the same species, which provides means to identify metabolites crucial for the interaction with the microbiota. In addition, the root exudates are affected by environment, particularly biotic factors ^10– 12^.

Other tools and approaches have been developed to dissect the mechanisms of communication between plants and their microbiome. This review will summarize recent progress in the identification of metabolites involved in plant–microbe interactions, provide a set of the most important open questions, and propose ways that these can be addressed and answered.

## Metabolites involved in communication between plants and root microbiota

The metabolites shaping plant microbiota belong to diverse classes. For example, the phenolic compounds coumarins are found across a wide variety of plant species and are relatively abundant in the rhizosphere where they have a well-characterized role in iron acquisition. However, two recent studies have independently shown that coumarins also play a key role in modulating root microbiome composition. Specifically, Stringlis *et al*. ^7^ showed that coumarin-deficient Arabidopsis mutants recruit a different set of taxa to their rhizosphere microbiome. This seems to be partially mediated via strain-specific antimicrobial effects because one particular coumarin, scopoletin, exerts toxicity against two fungal pathogens but not against two commensal bacteria. Using synthetic community (SynCom) inoculations, Voges *et al*. ^9^ show that the abundance of a *Pseudomonas* strain is significantly higher in coumarin-deficient Arabidopsis mutants compared with wild-type plants. The mechanistic basis of this phenomenon seems to involve redox-mediated microbial toxicity because the growth of this particular *Pseudomonas* strain is strongly inhibited by the hydrogen peroxide (H _2_O _2_) generated via another specific coumarin, sideretin ^9^.

Several decades of research have shown that benzoxazinoids, belonging to indole-derived metabolites, are key molecules conferring resistance against insect pathogens of maize ^13^. Recently, a suite of publications have analyzed how benzoxazinoids affect the maize microbiome. Using chemotaxis assays, Neal *et al*. ^14^ showed that DIMBOA recruits a growth-promoting *Pseudomonas* strain into the rhizosphere. Furthermore, a recent study showed that plants with mutated benzoxazinoid biosynthesis recruit altered microbiomes by using an approach that correlates liquid chromatography–mass spectrometry (LC-MS) metabolite profiling with operational taxonomic unit (OTU) sequencing data to reveal that benzoxazinoids stimulate the abundance of Methylophilaceae bacteria while repressing the abundance of Xanthomonadaceae ^15^. Intriguingly, the benzoxazinoid-mediated shifts in microbiome composition can also influence subsequent generations of plants ^16^, positioning these molecules as key agents in plant-soil feedback.

Another indolic compound, camalexin, is a well-characterized phytoalexin previously shown to exert fungal toxicity in leaves; however, a recent study also showed that it can modulate the functionality of root-associated microbial strains ^8^. Plant–microbe interaction assays show that camalexin plays a key role in modulating the effectiveness of growth-promoting bacteria because camalexin-deficient Arabidopsis mutants are unable to receive the growth benefits that mutualistic strains confer on the wild-type plants. Furthermore, microbial growth assays show that camalexin exerts selective toxicity against distinct bacterial strains isolated from plant roots ^8^.

Triterpenes are a class of secondary metabolites with incredible structural diversity. Unusually for plants, many of the enzymes catalyzing triterpene biosynthesis are transcribed from a set of gene clusters resembling bacterial operons. Recent work from Huang *et al*. ^17^ showed that triterpenes play a key role in modulating the Arabidopsis bacterial root microbiota. Growth assays of isolated strains show that purified triterpenes can stimulate the proliferation of an *Arenimonas* strain but that they inhibit the growth an *Arthrobacter* strain. Furthermore, detailed biochemical work revealed that certain strains isolated from field-grown plants possess the enzymatic machinery to use triterpenes as a carbon source ^17^.

Besides secondary metabolites, other components of plant root exudates (for example, aromatic organic acids) play a role in rhizosphere microbial community assembly. Zhalnina *et al*. ^6^ showed that *Avena barbata* secretes different metabolite profiles during development stages. This chemical succession together with bacterial substrate preference for consumption of aromatic organic acids (nicotinic, shikimic, salicylic, cinnamic, and indole-3-acetic) and amino acids contributes to the pattern of microbial community assembly ^6^. Correspondingly, rhizosphere bacteria encode a higher number of transporters for organic acids and amino acids in their genome ^18^.

Interestingly, the metabolite composition of root exudates can be influenced systemically and specifically by different bacterial strains ^12^. In a tomato split root system, 53 to 75% of metabolic features in LC-MS analysis of exudates of systemic roots were significantly changed after treatment of the local root with three different bacterial communities. Abundances of 93 metabolites were specifically affected by microbial treatment. Most of the regulated metabolites belonged to acylsugars, which have not been detected in roots or exudates before, or hydroxycinnamic acid conjugates ^12^. The analysis also revealed that the systemic changes in exudates are most likely transmitted through azelaic acid, identifying another plant metabolite important for shaping of root microbial community. Similarly, infection with a foliar pathogen *Pseudomonas syringae* pv. *tomato* resulted in alteration of at least 50 metabolites in Arabidopsis root exudates ^10^. The metabolic response was characterized by an enrichment of long-chain carbon compounds at the expense of short-chain compounds. Furthermore, preconditioning of soil using a mixture of these long-chain metabolites elicited improved pathogen resistance of subsequent plant generations, mediated via microbiome composition ^10^. This finding provides proof of concept for targeted bio-control strategies.

## Natural variation in metabolites shaping plant–microbe interactions

The discovery of such variety of metabolites affecting plant–microbe interactions in soil confirms the role of exudates for shaping the microbiome. As the secondary metabolite composition of different plant genotypes varies widely, approaches exploiting natural variation seem to be promising for identifying further components of rhizosphere signaling. The communication between plants and microbes occurs on multiple planes, and host genotype can clearly mold the associated microbiome communities ^3, 4^. These genetic variations in metabolic traits have recently been shown to influence the recruitment of microbial communities and also the microbial activity in rhizosphere soil, which overall have consequences for adaptations and host fitness.

An investigation of whether root exudate composition is genetically determined on a metabolic level in *Arabidopsis thaliana* was conducted by Mönchgesang *et al*. ^19^. The fundamental metabolites exhibiting natural variation in the exudates of the Multiparent Advanced Generation Inter-Cross (MAGIC) population were observed to be various glycosylated and sulfated compounds ^19^. When the metabolic profiles from the exudate metabolome were clustered, the 19 *A. thaliana* parental lines of the MAGIC population illustrated clear genetic variations. Similar conclusions were drawn when the health-promoting secondary metabolites (glucosinolates, phenolic acids, and flavonoids) and the sulfur and water availability in six different *Moringa oleifera* ecotypes were investigated ^20^. The differences observed in secondary metabolite content and composition laid the foundation for ecotype recommendations for intensive cultivation ^20^.

Given the importance of plant–microbe interactions for plant performance, to understand whether and how natural variation affects microbiome composition is of great importance. A genome-wide association study (GWAS) to determine how plants control their leaf microbiome used 196 Arabidopsis accessions and identified several candidate single-nucleotide polymorphisms ^21^. These were localized in genes responsible for cell wall synthesis, defense response, and kinase activity, which possibly contribute to the variation in the detected foliar microbiome composition. The composition of root microbiomes from the same genotype panel revealed that they differ significantly from the leaf microbiomes and that the host exerts a larger effect on fungal communities than on bacterial ones ^22^. Genes potentially affecting composition of root microbiome identified in a GWAS are involved in root development, vasculature, cell wall integrity, and immunity ^22^. These reports clearly demonstrate that plants affect the taxonomic composition of their microbiome and that the control is complex and involves multiple processes.

Accordingly, research into the microbiome composition instigated further investigations into how natural variation potentially affects the recruitment and assembly of plant growth-promoting microbes. In 2015, Haney *et al*. found wild accessions of *A. thaliana* to vary in their ability to influence the root-associated bacterium *Pseudomonas fluorescens*, affecting the hosts’ health ^23^. The accessions varied in the ability to support growth of *P. fluorescens* WCS365. Interestingly, accessions that negatively affected some *P. fluorescens* strains in the rhizosphere were not able to profit from plant growth-promoting effects of other *Pseudomonas* strains. So it seems that the compatibility within accessions represents evolutionary pressure, limiting the strains’ growth within the Arabidopsis rhizosphere ^23^. Thus, the plant genotype can promote its own health by affecting the microbiome; for example, in the presence of beneficial *P. fluorescens* and the pathogen *Fusarium oxysporum*, Arabidopsis genotypes that assist in rhizosphere colonization by *Pseudomonas* showed a selective advantage. However, biotic stress and compatibility with beneficial microbes may also be detrimental factors for the host fitness, as some growth-promoting bacteria, such as *Pseudomonas* strains CH229 and CH267, induce susceptibility to pathogenic bacteria ^23^.

Assessing the magnitude of the plant genotype effect versus the role played by the environment, Thiergart *et al*. ^24^ report that across large spatial planes the location and soil have a greater influence on the composition of the root microbiota than the host genotype of *A. thaliana*. Reciprocal transplants between two widely separated *A. thaliana* populations IT1 and SW4 (Italy and Sweden, respectively) demonstrated that the bacterial and fungal assemblages on the root interface are differentially regulated by edaphic factors and climatic conditions. Thus, here soil seems to be the primary factor exerting influence on the root microbiota, and the genetic variation provides little in comparison ^24^.

A pioneering study deciphered the influence of plant natural variation on the biological activity of microbial soil aryl-sulfatase activity ^8^. The analysis revealed more than a 10-fold difference of sulfatase activity in soil from 172 *A. thaliana* ecotypes, and a GWAS was performed to identify genes affecting the microbial community of the rhizosphere. Detailed analysis of the first selected candidate gene resulted in the identification of a new cytochrome P450 enzyme in camalexin biosynthesis and revealed a new role of camalexin in plant–microbe interactions ^8^. This again points to the importance of plant immune reactions in shaping their microbiome.

## New approaches to identify metabolites critical for plant–microbe interactions

The compounds discussed above provide the first insights into how plants interact with root microbes. However, these insights are far from complete because the composition of root exudates is complex and changes depending on development stage, plant species, soil type, and other biotic or abiotic factors. To get a deeper insight into plant–rhizosphere bacteria interactions, new technologies have been developed. An exometabolomics (study of extracellular metabolites) approach, especially untargeted, provides detailed information about the complexity of root exudates and how they are affected by microbiota, enabling studies on bacterial substrate preferences from a mixture of exuded metabolites ^25^. By comparing exometabolite data, it is possible to find key compounds modulating plant–bacteria interactions ^26^. The exometobolomic methodology can also be used to dissect cross-feeding between plants and root microbes when root exudates serve as the sole carbon source for cultivation of the rhizosphere bacteria ^25^. This new approach thus allows researchers to investigate bacterial substrate preference from hundreds of metabolites at the same time, which mimics the real rhizosphere environment.

SynComs are an excellent tool to study plant–microbe interactions as defined bacterial communities can be assembled from large microbial collections ^27^. SynComs can be designed to elucidate and predict outputs caused by specific characteristics of bacterial consortia. Pioneering work using SynCom approaches showed that the defense phytohormone salicylic acid (SA) modulates bacterial colonization of the roots of Arabidopsis ^28^. Removal of all defense phytohormone signaling pathways (SA, jasmonic acid, and ethylene) results in an abnormal root microbial profile, which may lessen survival in nature. In that study, a SynCom of 38 bacteria strains was designed according to representation of family OTU categories and included strains differentially enriched in wild-type versus defense hormone mutants. This specific SynCom revealed that the absence of SA prevented the mutants from excluding bacteria that normally do not colonize wild-type ^28^. Another study took advantage of SynComs to investigate links between phosphate starvation response, immune system function, and root microbiome assembly, which act simultaneously in nature ^29^. That study showed the importance of genes controlling the phosphate starvation response for composition of root microbiome and revealed a novel function of the transcription factor PHR1 as a direct regulator of a subset of immunity genes, including genes for synthesis of sulfur-containing secondary compounds, the glucosinolates ^29^. Furthermore, in an attempt to obtain a simplified but stable bacterial SynCom, Niu *et al*. ^30^ identified seven strains, which represented three of the four most dominant phyla in maize roots. This simple SynCom remained stable over long periods of time and was beneficial for the plant performance. The key finding was that one specific strain is necessary for stability of the community because if that keystone strain is removed, then the community diversity collapses ^30^. The design of SynComs can be assisted by plant–bacterium binary-association assays that may serve as bases for machine learning approaches to predict the plant phenotypes after inoculation ^31^. This approach, together with hydroponic gnotobiotic systems, creates controlled and reproducible conditions to clarify plant–microbe interactions, providing a pathway to predict the functionality of complex bacterial consortia and plant phenotype. Such use of SynComs can be further refined by using microfluidics systems ^32^, allowing, for example, the use of sensors for detection of specific metabolites and their dynamic responses to the microorganisms.

## Conclusions

The recent breakthroughs in identifying rhizospheric signals between plants and microbes clearly accentuate that the metabolites exuded by roots enable plants to shape their microbiome. They also reveal that the metabolites form a dynamic and complex signaling network that also includes microbe-derived compounds. The challenges now are to identify further components of the signaling network and to understand the mechanisms by which the alterations in microbiome composition and functions, such as nutrient availability or pathogen suppression, are achieved. The new approaches summarized above will certainly contribute to addressing this challenge. Exploitation of natural variation and metabolomics will help to identify further components of the signaling networks and, in combination with SynComs, will enable questions on the function of individual rhizosphere metabolites to be answered. The interactions between microbial community members require more attention and the contribution of physicochemical soil properties. Ultimately, understanding how plants shape their microbiome will enable the development of biofertilization strategies using specific signals to recruit specific beneficial microbes for a given soil and environment.

## Abbreviations

GWAS, genome-wide association study; LC-MS, liquid chromatography–mass spectrometry; MAGIC, Multiparent Advanced Generation Inter-Cross; OTU, operational taxonomic unit; SA, salicylic acid; SynCom, synthetic community
